# Parenchymal Sparing Resection: Options in Duodenal and Pancreatic Surgery

**DOI:** 10.3390/jcm10071479

**Published:** 2021-04-02

**Authors:** Ugo Marchese, Stylianos Tzedakis, Einas Abou Ali, Olivier Turrini, Jean-Robert Delpero, Romain Coriat, David Fuks

**Affiliations:** 1 Department of Digestive, Hepatobiliary and Pancreatic Surgery, Cochin Hospital, AP-HP Centre, 27 rue du Faubourg Saint-Jacques, 75014 Paris, France; stylianos.tzedakis@aphp.fr (S.T.); david.fuks@aphp.fr (D.F.); 2 Faculté de Médecine, Université de Paris, 75006 Paris, France; einas.abouali@aphp.fr (E.A.A.); romain.coriat@aphp.fr (R.C.); 3 Gastroenterology and Digestive Oncology Unit, Cochin Hospital, AP-HP Centre, 27 rue du Faubourg Saint-Jacques, 75014 Paris, France; 4 Faculté de Médecine, Université d’Aix Marseille, 13005 Marseille, France; turrinio@ipc.unicancer.fr (O.T.); jrdelpero@gmail.com (J.-R.D.); 5 Department of Surgical Oncology, Paoli Calmettes Institute, 232 Boulevard de Sainte Marguerite, 13009 Marseille, France

**Keywords:** pancreatic tumor, pancreatectomy, pancreatic sparing surgery, long-term functional outcomes, ampullectomy, central-pancreatectomy, duodenectomy, uncus resection, enucleation

## Abstract

Parenchymal sparing duodenal and pancreatic resection are safe procedures in selected patients with the aim to reduce endocrine and exocrine long-term dysfunction. When the tumor is benign or borderline malignant, this appears to be a good option for the surgeon, associated with low rates of severe surgery-related early postoperative complications and low in-hospital mortality. This mini review offers comments, tips and tricks, and a review of literature concerning those different options with specific illustrations in order to clarify their indication.

## 1. Introduction

The frequency of intraductal papillary mucinous neoplasms (IPMNs), mucinous cystic neoplasms (MCNs) and pancreatic neuroendocrine tumors (pNETs), representing benign and borderline pancreatic tumors, has been increasing with modern routine abdominal imaging. While most of pNETs are represented by localized well-differentiated forms with a reported five-year survival rate of 70–90% [1,2,3], intraductal papillary mucinous neoplasms (IPMNs) and mucinous cystic neoplasms (MCNs) can shift toward pancreatic malignancy and should be detected and treated before transition to invasive cancer. Solid pseudopapillary neoplasm of the pancreas is a rare tumor that could be treated with limited resection, given its low malignant potential.

Standard pancreatic resection with lymphadenectomy, including pancreaticoduodenectomy and distal pancreatectomy, is the standard treatment for pancreatic tumors [4]. However, these extensive procedures are associated with a significant morbidity, mortality, and an impaired pancreatic exocrine (9–60%) and endocrine function (7–35%) according to both the extent and the side of pancreatic resection [5,6].

Benign and premalignant tumors of the pancreas represent a challenge for pancreatic surgeons and different pancreas-sparing procedures, such as pancreatic sparing partial or total duodenectomy, surgical ampullectomy, tumor enucleation (TE), uncus resection and central pancreatectomy (CP) (Table 1), have been proposed for reducing the incidence of postoperative pancreatic endocrine and exocrine insufficiency [7].

Regardless, the decision to perform surgical resection in patients with benign and borderline premalignant pancreatic tumors should be based on the recommendation of a multidisciplinary team with substantial expertise in both pancreatic disease and pancreatic surgery. Nevertheless, it is of paramount importance to carefully weight the oncological risk along with the effort to achieve a substantial risk reduction of both long-term exocrine and endocrine postoperative failure.

Given the increased risk of postoperative complications of some procedures (i.e., pancreatic middle segment resection (PMSR) or pancreatic sparing duodenectomy), these interventions require particular expertise in pancreatic surgery and patients should be preferentially referred in tertiary pancreatic centers. 

This mini review aims at presenting an overview of the different surgical procedures preserving the pancreatic parenchyma in the treatment of premalignant pancreatic neoplasms as IPMN, pNETs, or pancreatic cystadenomas.

## 2. Enucleation

### 2.1. Indications

Among pancreatic sparing strategies, enucleation is indicated for superficial tumors that did not involve the main pancreatic duct (MPD). In this manner, enucleation was first performed in the 1960s; currently, neuroendocrine tumors (particularly insulinoma) and branch-duct intraductal papillary mucinous neoplasms (IPMNs) are the more frequent indication of enucleation [8]. Current surgical principles consider that enucleation is only suitable for tumors less than 2 cm in diameter, and the distance between the tumor and main pancreatic duct needs to be >3 mm. However, this point has not been validated by large clinical databases, and the research using small sample data is also scarce. The application value of enucleation for pNETs still need to be determined [9].

### 2.2. Imaging Required

**Figure 1:**
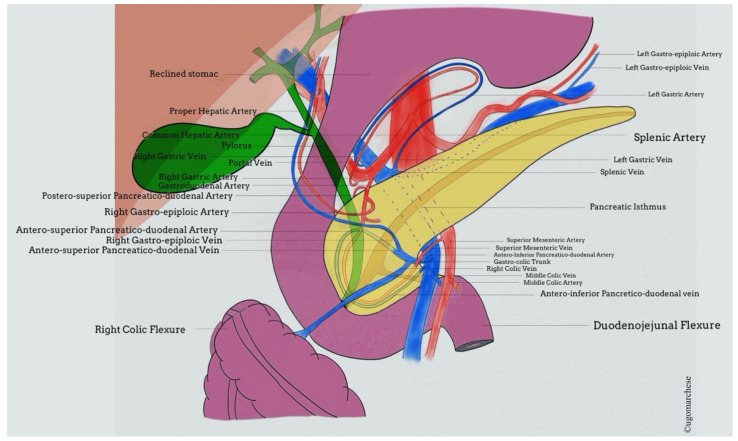
Pancreas anatomy: vascularization and relationship between common bile duct and main pancreatic duct.

Given criteria for enucleation, preoperative imaging needs to assess location of tumor and its relationship with main pancreatic duct and accessory duct identification (Figure 1). As MRI (magnetic resonance imaging) is the most relevant imaging exam to assess pancreatic tumors and anatomy of both MPD and common bile duct (CBD), only patients who had a recent preoperative MRI should be considered for enucleation.

### 2.3. Surgical Tips

Before surgery, patients planned for an enucleation must be informed that a standard resection could be finally achieved according to intraoperative findings. During the surgery, tumor should be localized within the pancreatic parenchyma using intraoperative ultrasonography (IOUS). IOUS in combination with intraoperative palpation by an experienced operator has been shown to achieve excellent detection rates of 80–100% [8,10].

**Figure 2:**
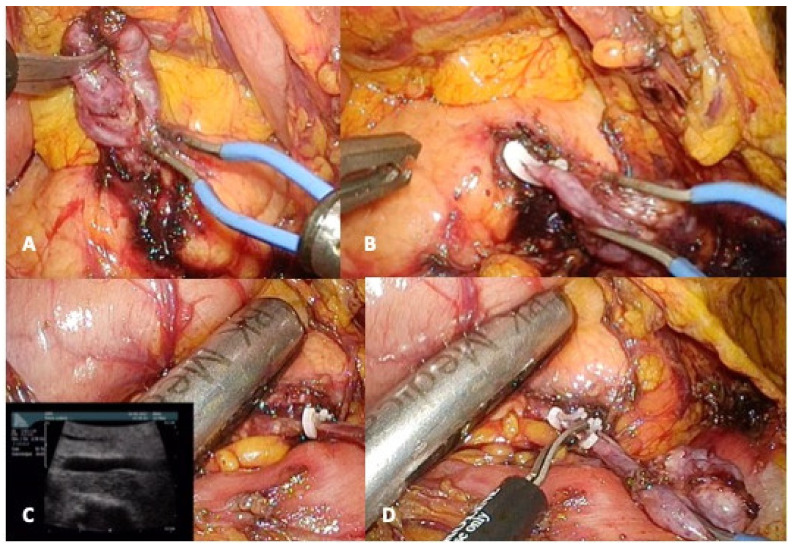
Laparoscopic enucleation for intraductal papillary mucinous neoplasms (IPMN). (**A**) Identification of the communicant branch duct; (**B**) Secured clip at the origin of the communicant branch duct; (**C**) intraoperative ultrasonographic control; (**D**) Section of the communicant branch duct.

Indeed, when the enucleation of a deep tumor is planned, a sphincterotomy followed by the insertion of a stent in the MPD could be discussed prior to surgery to facilitate intraoperative identification of surgical margin. However, its role remains unclear to avoid MPD injury and post-operative pancreatic fistula (POPF) and is correlated with a non-negligible risk of acute pancreatitis [11]. For all these reasons, preoperative stent cannot be proposed as a preoperative routine procedure (Figure 2).

If a branch-duct IPMN enucleation is planned, the surgeon must intraoperatively identify the communicant duct in order to (a) have it frozen section examination (as well as the tumor itself to not ignore a high-grade dysplasia or invasive IPMNs that must require a standard resection), and (b) allow its elective ligation to reduce the POPF risk.

As for any pancreatic surgery, a drainage should be placed in the enucleation zone to permit an optimal drainage in case of POPF. We highlight that an enucleation achieved in the anterior face of the pancreatic head is difficult to optimally drain as the anatomical position often lead to an “egg cup” with the risk of pancreatic juice stagnation.

### 2.4. Results

Despite the fact that several studies showed that enucleation is simpler and allows shorter operative time, faster recovery, and reduced postoperative complications, we need to keep in mind that the proximity of the surgical section with the MPD is associated with an increased risk of POPF. Indeed, as enucleation induces parenchyma incision and, occasionally, deep pancreas opening, it exposes patients to POPF by unknown MPD injury or weakening, especially if thermo-coagulation has been used too closely. Consequently, several reports have shown that enucleation leads to high rate of POPF than does standard pancreatectomy (around 43–45%), but with a “toward zero” mortality. Predictive factors of POPF after enucleation have already been reported, such as age, body mass index, distance from the main pancreatic duct (≤3 mm), cystic morphology, and history of acute pancreatitis [12]. These factors are not a contraindication for enucleation but could help pancreatic surgeons to inform patients about possible prolonged postoperative courses, counterbalanced by the very low risk of developing diabetes mellitus and/or steatorrhea. The pancreatic location of the enucleation (head/uncinate process/body/tail) seemed to be a relevant factor of POPF [13]. In case of prolonged high output POPF after branch-duct IPMN enucleation, a pancreatic stent could be placed to cover the communicant duct and solve the fistula.

Interestingly, minimally invasive enucleation has been recently adopted since it does not require any reconstructions. While the level of evidence remains poor, the literature suggests that both surgical approaches provide similar short- and long-term results.

## 3. Uncus Resection

### 3.1. Indications

The uncinate process is defined by a portion of the head of the pancreas that hooks around posterior to the superior mesenteric vessels but is actually difficult to delimit from the proper head parenchyma on preoperative imaging and during surgery [13].

If IPMN remains the most frequent indication of duodenum preserving partial pancreatic head resection, other low-malignant pancreatic neoplasm located in the uncus should also be considered as potential candidates.

### 3.2. Imaging Required

**Figure 3:**
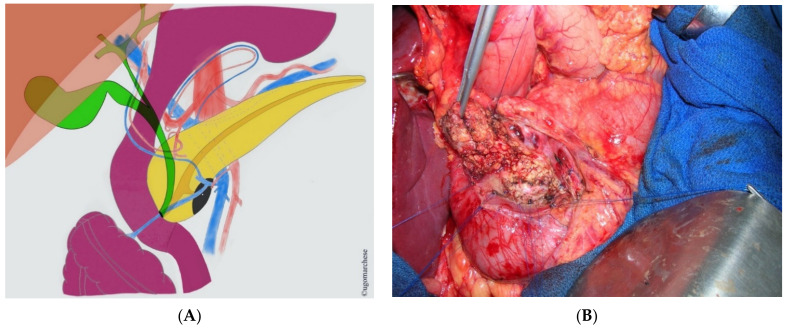
Uncus resection: (**A**) Schema; (**B**) Surgical view.

Exact location of MPD and CBD, as well as their relationship into the pancreatic head, is mandatory (Figure 3). Wirsungo-MRI with 3D reconstruction should be performed systematically before resection in order to identify potential pitfalls as an eventual pancreas divisum and accessory papilla location, an invasive duodenal lesion or biliary tract invasion that can be suspected by jaundice. Those ascertainments should conduct to reconsider pancreatic head preserving partial or total duodenectomy to the benefit of standard resection.

### 3.3. Surgical Tips

As for patients planned for enucleation, patients planned for an uncus resection must be informed that a standard resection could be finally achieved according to intraoperative findings. The same way, as mentioned in pancreatic enucleation, tumor should be localized within the pancreatic parenchyma using IOUS in combination with intraoperative palpation by an experienced operator. Interestingly, the uncinate process can be approached from both right and left sides around the superior mesenteric vessels after section of the Treitz ligament [14]. A special attention should be paid to the duodenum vascularization. Indeed, uncus resection could lead to duodenum devascularization below the genu inferius that should be identified intraoperatively. In such a situation, a duodenal resection or a pancreatoduodenectomy should be achieved.

### 3.4. Results

As a duodenum preserving partial pancreatic head resection, uncus resection is a safe surgical procedure associated with a low frequency of severe surgery-related early postoperative complications and low in-hospital mortality in high volume centers. This procedure reduces endocrine and exocrine dysfunction of the pancreas obviously when the tumor does not involve the duodenum itself. Uncus resection represents the most frequent duodenum preserving partial pancreatic head resection procedure that may theoretically reduce the risk of postoperative cholangitis compared to standard pancreatoduodenectomy (PD). A systematic review was conducted to evaluate the outcome of patients after parenchyma-sparing pancreatic head resection in the treatment of premalignant cystic neoplasms and pNETs. The advantages of duodenum-sparing pancreatic head resection are low surgery-related morbidity but steel around 9% of severe complications [15].

## 4. Central Pancreatectomy

### 4.1. Indications

For lesions that are located in the neck and proximal body of pancreas for which enucleation is not possible, central pancreatectomy can represent an interesting alternative particularly, if the remaining distal pancreas is more than 5 cm in length, without any adjacent organ or gastro duodenal artery invasion [16].

Central pancreatectomy is often performed in young women (59–73%) (47–52 years) and mainly for NET. Less frequently, it was indicated for IPMN, for mucinous cystadenomas, and, more recently, for solid and pseudo-papillary tumors [17]. The feasibility of central pancreatectomy was also demonstrated for some particular malignancies of the pancreas, such as metastases of other malignancies.

Given the morbidity of this procedure, central pancreatectomy should be reserved for patients at relative low risk of POPF even though contraindications include fatty infiltration of the pancreas and high risk of pancreatic fistula, age > 70 years, length of the residual distal pancreas < 5 cm, patients with pre-existing diabetes, and/or use of therapeutic anticoagulation medications [18,19].

### 4.2. Imaging Required

Arterial phase CT (computed tomography) scan and/or MRI are mandatory in order to identify margin around gastroduodenal artery, common hepatic artery, and splenic artery.

### 4.3. Surgical Tips

**Figure 4:**
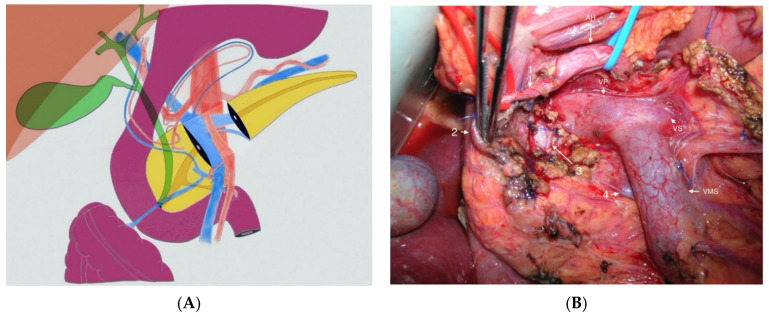
Central pancreatectomy: (**A**) Schema; (**B**) Central pancreatectomy extended to the right for isthmic pancreatic tumor. AH: hepatic artery; VP: portal vein; VS: splenic vein; VMS: superior mesenteric vein; 1: Gastroduodenal artery; 2: Superior pancreaticoduodenal artery; 3: common bile duct; 4: Inferior pancreaticoduodenal vein.

Conservation of splenic artery is usually feasible (Figure 4). Pre- or intraoperative clearance of gastroduodenal artery is recommended. If necessary, pancreatic section can be extended to the right but the gastroduodenal artery should not be sacrificed and should make discuss conversion in a standard PD. Pancreatojejunostomy are more frequent than pancreatogastrostomy, even if data are to limited to recommend one technique against another.

Drainage of central pancreatectomy is not easy. Indeed, the pancreatic head prevent an efficient right-sided drainage; and, if a left-sided drainage is decided, it should be placed backward the left colic flexure (and not above it) to not be exposed to a rapid postoperative drain displacement.

### 4.4. Results

Although the number of publications reporting central pancreatectomy has increased in recent years, this procedure represents less than 5% of total pancreatic resections, even in high-volume centers. Currently, the benefits of central pancreatectomy over distal pancreatectomy (DP) remain controversial.

The potential long-term functional benefits of CP should be regarded in balance with the widely reported high morbidity rates. Thus, in few series reporting at least 100 patients with central pancreatectomy, the overall morbidity and POPF rates were 58–72% and 44–63%, respectively, while the incidences of endocrine and exocrine insufficiencies were 4–7.5% and 0–6%, respectively [17]. This high rate of postoperative complications is the consequence of both two potential locations of pancreatic leakage and because pancreatic parenchyma is mostly soft in benign or borderline malignant lesions. A recent meta-analysis suggested that central pancreatectomy allows better preservation of pancreatic functions but also increases rates of complications, including severe complications when compared to distal pancreatectomy [18].

Proper selection of patients for central pancreatectomy is crucial not only to maximize the functional benefits but also to mitigate the effects of potential complications. Central pancreatectomy should be considered as an alternative to distal pancreatectomy in young patients with no associated diabetes mellitus and no significant comorbidities with a functionally meaningful distal remnant pancreas. Long-term postoperative complication as endocrine and exocrine pancreatic insufficiency is significantly lower than after standard pancreatic resection and estimated around in 5.0% and 9.9% respectively of patients.

## 5. Ampullectomy

### 5.1. Indications

Very few indications of surgical ampullectomy remains at the era of endoscopic ampullectomy, which is successful upfront in 61% to 92% of cases. Surgical attempts to remove the ampullary region should be considered in first intention only when the ampullary adenoma is technically non-accessible (medical history of by-pass procedure, duodenal diverticulosis) or in case of CBD extension. Intraductal involvement, jaundice and occult malignant foci in the resected specimen are associated with a lower rate of margin negative resection either in endoscopic than in surgical ampullectomy [20]. Recurrence rates after endoscopic ampullectomy are 0% to 33%, and, in these cases, the surgeon should consider a surgical ampullectomy, except if the initial endoscopic resection revealed an invasive neoplasm.

In summary, if the center’s endoscopists are very skilled and experienced, the pancreatic surgeons will very rarely have to perform an ampullectomy. Indeed, endoscopists are pushing the limits of endoscopic resection as ampullectomy could be associated with endoscopic radiofrequency to eliminate a tumor residue, and could be repeated to insure tumor clearance.

### 5.2. Imaging Requires

Wirsungo-MRI should be performed before resection in order to identify potential pitfalls as an eventual pancreas divisum and accessory papilla location, an invasive duodenal lesion or biliary tract invasion that can be suspected by a jaundice. These ascertainments should conduct to reconsider surgical or endoscopic ampullectomy to the benefit of standard resection. Endoscopic ultrasound with a rigorous analysis of anatomy and tumor extension remains an essential tool in the planning step of pancreatic and duodenal sparing resections, in combination to MRI.

### 5.3. Surgical Tips

**Figure 5:**
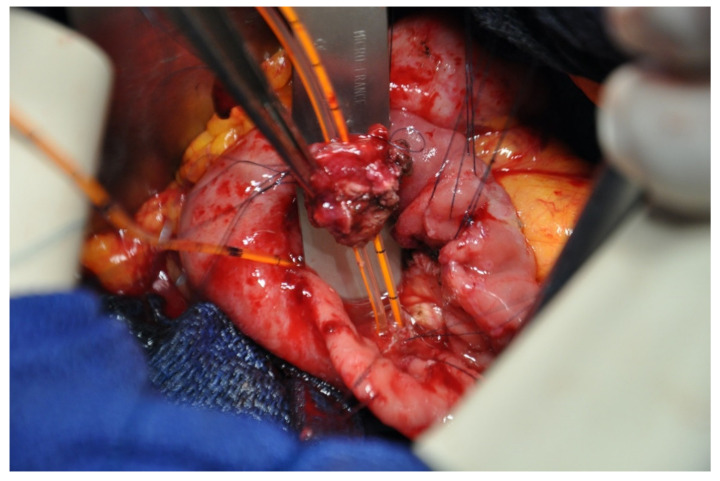
Ampullectomy (surgical view).

Intra operative frozen section biopsy has to be carried out to confirm tumor negative status of the cut margin on MPD and CBD (Figure 5). If frozen section biopsy on MPD reveals positive margin, a PD should be performed. Therefore, patients planned for an ampullectomy must be informed that a PD could be finally achieved according to intraoperative findings. In case of isolated intraepithelial neoplasia on the CBD margin, the resection could be extending to the intrapancreatic CBD. In this particular indication, a choledoco-jejunostomy should be performed [21,22]. As described in duodenectomy, external drainage of MPD and CBD may facilitate the postoperative courses, but no evidence about its benefit are available.

### 5.4. Results

While endoscopic ampullectomy is currently the best strategy for the treatment of ampullary neoplasms with absence of or low-grade dysplasia, transduodenal ampullectomy represents a surgical option. A recent study comparing endoscopic and surgical approaches found that endoscopic ampullectomy had equivalent efficacy, but lower morbidity compared to surgical approach (18% versus 42% for endoscopic and surgical approaches, respectively [23]. The literature on transduodenal ampullectomy remains limited. The largest series of this procedure was published by the Heidelberg group and included 83 patients [20] and a recent series published by Nappo G et al. included 36 patients. In this non-comparative study, the morbidity was almost 45%, with 13.9% of severe complication without any postoperative death.

## 6. Pancreatic Head Preserving Partial or Total Duodenectomy

### 6.1. Indication

Even if pancreatoduodenectomy (PD) is the gold standard for resection of duodenal tumors, total duodenectomy with pancreatic head preservation could be an option for duodenal gastrointestinal stromal tumours (GISTs), large or multifocal duodenal carcinoids or when duodenal polyps occur as a part of a familial adenomatous polyposis syndrome [24,25].

In this situations, endoscopic resection, endoscopic mucosal resection (EMR) or endoscopic submucosal dissection (ESD) should be firstly attempted. However, in patients with familial adenomatous polyposis syndrome, total duodenectomy allows a pancreas preserving complete removal of the high-risk duodenal mucosa. It may be a secured option especially in case of peri ampullary lesion with a cumulated 10% risk of periampullary adenocarcinoma at the age of 60 [26].

Finally, as the duodenum is the most dangerous location in which to perform ESD, these endoscopic procedures may be discuss from a technical perspective and summited to multidisciplinary board evaluation. Given the high rate of local recurrence for polyps larger than 20–30 mm (upwards of 25% in case of piecemeal resection), the surgical option could be considered. In this situation, partial or total duodenectomy preserving the pancreatic head is an interesting alternative to PD associated with less morbidity and no loss of pancreatic parenchyma.

### 6.2. Imaging Required

Pancreatic MRI with specific analysis of main pancreatic duct should be performed before resection in order to identify potential pitfalls as pancreas divisum, accessory papillary, invasive duodenal lesion, or biliary tract invasion. These ascertainments should lead the surgeon to reconsider pancreatic head preserving partial or total duodenectomy to the benefit of standard resection.

### 6.3. Surgical Tips

**Figure 6:**
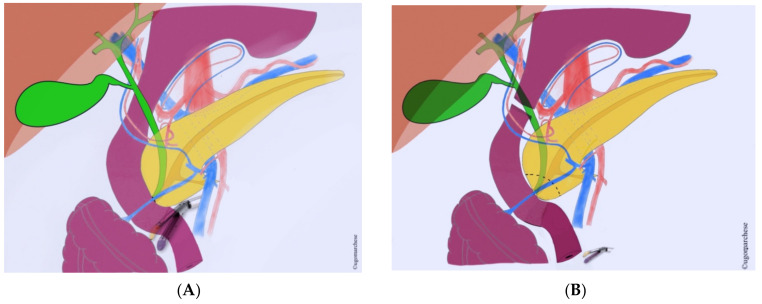
Duodenal sparing resection: (**A**) Partial distal duodenectomy. (**B**) Total duodenectomy with ampullectomy.

During the partial duodenectomy, intraoperative endoscopy aims controlling the position of linear staplers’ line on the duodenum under direct vision, accurately evaluating the longitudinal margin due to dissection with a linear stapler and eventually identifying the location of duodenal papillary. Even if duodenal papillary seems free, a systematic intraoperative cholangiography is recommended before stapling (Figure 6).

Reconstruction after total duodenectomy often needs external drainage of main pancreatic duct (MPD) and common biliary duct (CBD) without formal evidence of benefit on postoperative morbidity. As previously mentioned, any intraoperative deviation from original plan, it should be considered to switch from pancreatic head, preserving partial or total duodenectomy to the benefit of standard resection.

### 6.4. Results

**Figure 7:**
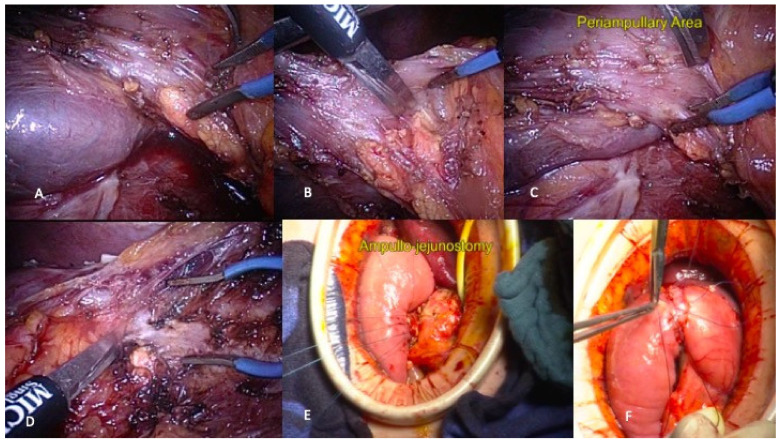
Laparoscopic total duodenectomy. (**A**) Kocher maneuver; (**B**) Duodenopancreatic division; (**C**) Identification of periampullary area; (**D**) Section of the ampulla; (**E**) Ampullo-jejunostomy by minilap incision; (**F**) Vision at the end of the procedure.

Approximatively 50% of patients will experience significant complications, and almost 30% of them will develop severe complications, including pancreatic leakage, delayed gastric emptying, acute pancreatitis, wound infection, and/or intra-abdominal sepsis [26,27,28]. In selected cases, the laparoscopic approach can be considered as illustrated in Figure 7.

Nevertheless, pancreatic functions remain preserved, especially if the duodenal ampulla of Vater is conserved. Pancreatic preserving duodenectomy can be considered in benign and recurrent duodenal tumors involving neither the MPD nor the CBD.

## 7. Conclusions

Parenchymal sparing duodenal and pancreatic resection represent an interesting alternative to standard resection for benign and premalignant tumors with excellent long-term functional result. Despite a mandatory minimum morbidity and mortality, the benefit-risk balance of these limited resections should be carefully evaluated before and during surgical procedure.

## Figures and Tables

**Table 1:** Summarized information on duodenal and pancreatic parenchymal sparing resection.

Procedure	Indication	Contraindication	Preoperative Workup	Tips and Pitfalls	Results
Enucleation	pNETs BD-IPMNs MCNs	Invasive tumor Size > 2 cm Distance with MPD < 3 mm	MRI Endoscopic Ultrasound	Intraoperative ultrasonography Preoperative MPD stenting	POPF: 43–45% Toward zero mortality
Uncus resection	pNETs BD-IPMNs	Invasive tumor Size > 2 cm Distance with MPD < 3 mm	Wirsungo-MRI with 3D reconstruction Endoscopic Ultrasound	Intraoperative ultrasonography Preoperative MPD stenting Intraoperative cholangiography Consider left sided approach	Severe morbidity: 9% Toward zero Mortality
Central pancreatectomy	pNETs MCNs SPPT IPMNs	High risk of POPF >70 years old Residual pancreas < 5 cm Preoperative diabetes mellitus	CT scan with vascular reconstruction MRI Endoscopic Ultrasound	Gastroduodenal artery clearance mandatory Left Drainage backward left colic flexure	Morbidity: 58–72% 50% decrease of exocrine and endocrine insufficiency
Ampullectomy	No endoscopic access to ampulla CBD extention	Invasive tumor Jaundice (relative)	MRI Endoscopic Ultrasound	Systematic frozen section analysis on MPD and CBD External drainage of MPD and CBD	Morbidity: 42% (vs. 18 for endoscopy) Severe morbidity: 13.9%
Pancreatic head preserving duodenectomy	Duodenal GIST Large or multiple Carcinoid neoplasm Polyps larger than 20–30 mm Duodenal polyps in FAP syndrome	Invasive tumor Jaundice Pancreatic head invasion	MRI Endoscopic Ultrasound	Endoscopic intraoperative surgical margin control Intraoperative cholangiography	Severe morbidity: 30% Improved functional outcomes

pNETs pancreatic neuroendocrine tumors, BD-IPMNs branch duct intraductal papillary mucinous neoplasms, MCNs mucinous cyst neoplasms, SPPT solid and pseudo-papillary tumor, MPD main pancreatic duct, CBD common biliary duct, POPF post-operative pancreatic fistula, FAP familial adenomatous polyposis, MRI, magnetic resonance imaging, CT, computed tomography, GIST, gastro intestinal stromal tumors.

## Data Availability

Not applicable.
